# Quality of Care Before and After Mergers and Acquisitions of Rural Hospitals

**DOI:** 10.1001/jamanetworkopen.2021.24662

**Published:** 2021-09-20

**Authors:** H. Joanna Jiang, Kathryn R. Fingar, Lan Liang, Rachel M. Henke, Teresa P. Gibson

**Affiliations:** 1 Agency for Healthcare Research and Quality, Rockville, Maryland; 2 IBM Watson Health, Sacramento, California; 3 IBM Watson Health, Cambridge, Massachusetts; 4 IBM Watson Health, Rochester, New York

## Abstract

**Importance:**

Rural hospitals are increasingly merging with other hospitals. The associations of hospital mergers with quality of care need further investigation.

**Objectives:**

To examine changes in quality of care for patients at rural hospitals that merged compared with those that remained independent.

**Design, Setting, and Participants:**

In this case-control study, mergers at community nonrehabilitation hospitals in Federal Office of Rural Health Policy–eligible zip codes during 2009 to 2016 in 32 states were identified from Irving Levin Associates and the American Hospital Association Annual Survey. Outcomes for inpatient stays for select conditions and elective procedures were derived from the Healthcare Cost and Utilization Project State Inpatient Databases. Difference-in-differences linear probability models were used to assess premerger to postmerger changes in outcomes for patients discharged from merged vs comparison hospitals that remained independent. Data were analyzed from February to December 2020.

**Exposures:**

Hospital mergers.

**Main Outcomes and Measures:**

The main outcome was in-hospital mortality among patients admitted for acute myocardial infarction (AMI), heart failure, stroke, gastrointestinal hemorrhage, hip fracture, or pneumonia, as well as complications during stays for elective surgeries.

**Results:**

A total of 172 merged hospitals and 266 comparison hospitals were analyzed. After matching, baseline patient characteristics were similar for 303 747 medical stays and 175 970 surgical stays at merged hospitals and 461 092 medical stays and 278 070 surgical stays at comparison hospitals. In-hospital mortality among AMI stays decreased from premerger to postmerger at merged hospitals (9.4% to 5.0%) and comparison hospitals (7.9% to 6.3%). Adjusting for patient, hospital, and community characteristics, the decrease in in-hospital mortality among AMI stays 1 year postmerger was 1.755 (95% CI, −2.825 to −0.685) percentage points greater at merged hospitals than at comparison hospitals (*P* < .001). This finding held up to 4 years postmerger (DID, −2.039 [95% CI, −3.388 to −0.691] percentage points; *P* = .003). Greater premerger to postmerger decreases in mortality at merged vs comparison hospitals were also observed at 5 years postmerger among stays for heart failure (DID, −0.756 [95% CI, −1.448 to −0.064] percentage points; *P* = .03), stroke (DID, −1.667 [95% CI, −3.050 to −0.283] percentage points; *P* = .02), and pneumonia (DID, −0.862 [95% CI, −1.681 to −0.042] percentage points; *P* = .04).

**Conclusions and Relevance:**

These findings suggest that rural hospital mergers were associated with better mortality outcomes for AMI and several other conditions. This finding is important to enhancing rural health care and reducing urban-rural disparities in quality of care.

## Key Points

**Question:**

Are mergers and acquisitions associated with the quality of care at rural hospitals?

**Findings:**

This case-control study including 172 merged hospitals and 266 comparison hospitals that remained independent compared premerger to postmerger changes in in-hospital mortality for common conditions and complications for elective procedures. Adjusted for patient, hospital, and community characteristics, decreases in mortality among stays for acute myocardial infarction, heart failure, stroke, and pneumonia postmerger were greater at merged hospitals than at comparison hospitals.

**Meaning:**

These findings suggest that rural hospital mergers were associated with better mortality outcomes for certain conditions.

## Teaser

This case-control study examines changes in quality of care at rural hospitals that merged with or were acquired by other hospitals compared with hospitals that remained independent.

## Introduction

More than one-third of US community hospitals are located in rural areas, serving as the principal source of care for 60 million people, nearly 20% of the US population.^1,2^ For the past few decades, rural hospitals have faced declining populations, worsening economic conditions, and persistent shortages of clinicians,^3^ putting them at greater risk of closure than their urban counterparts.^4,5,6,7^ To avoid closure, merger and acquisition, hereafter referred to as *merger*, may be an option for sustaining rural hospitals and ensuring essential access to care for their communities.^8^ The number of mergers among rural hospitals has increased significantly since the mid-2000s, in parallel with the accelerating number of closures.^9,10^

Although mergers may enhance rural hospital survival, they may also have effects on quality of care. Prior studies on this topic tended to focus on urban hospitals, with the primary concern that consolidation could lead to increased market power. These studies were inconclusive on the association between hospital mergers and quality of care as measured by mortality, readmissions, complications, clinical processes, and patient experience.^11,12,13,14,15,16^ To our knowledge, only 1 recent study, by O’Hanlon and colleagues,^17^ specifically examined quality of care along with access to care and financial performance after rural hospital affiliation with health systems. The authors found significant improvement in hospital operating margins after system affiliation but no difference in patient experience and 30-day hospital-wide all-cause readmissions.^17^

Given that most rural hospitals are the only acute care hospital in the community, mergers would not necessarily lead to greater market consolidation. However, rural hospital mergers may increase market power through collective negotiation with payers. Mergers also could lead to reductions in service lines.^17^ A potential benefit of merger for small and isolated rural hospitals is that it may enable access to needed financial resources (eg, capital), clinical expertise (eg, specialized services), and new technologies (eg, electronic health records).^18^ Mergers may also offer opportunities for rural hospitals to join alternative payment models, such as accountable care organizations.^8^ Thus, rural hospitals may be able to provide higher quality of care after mergers.

The purpose of this study was to assess changes in inpatient quality of care for rural hospitals that merged or were acquired compared with rural hospitals that remained independent. We expanded on prior research by leveraging all-payer discharge databases to measure hospital quality in terms of mortality and complications. We used multiple data sources to identify mergers and used a difference-in-differences (DID) design to compare changes in quality between merged hospitals and independent hospitals.

## Methods

The Healthcare Cost and Utilization Project (HCUP) databases used in this case-control study are consistent with the definition of limited data sets under the Health Insurance Portability and Accountability Act Privacy Rule and do not constitute research involving human participants requiring review by an institutional review board or participant informed consent. This study follows the Strengthening the Reporting of Observational Studies in Epidemiology (STROBE) reporting guideline for observational studies.

### Study Population and Data

We included community, nonrehabilitation, non–long-term, general acute care hospitals in rural zip codes defined as eligible to receive funding by the Federal Office of Rural Health Policy.^19^ We identified hospital mergers from 2009 to 2016 using data from Irving Levin Associates and the American Hospital Association’s (AHA) Annual Survey (eAppendix 1 in the Supplement). We selected rural comparison hospitals in the same states as merging hospitals that reported no system membership to the AHA throughout the study period. Information on hospital attributes were obtained from the AHA, and community characteristics were drawn from the American Community Survey and Area Health Resources Files.

To measure quality of care, we used all-payer discharge data from the 2008 to 2018 HCUP State Inpatient Databases (SID)^20^ to identify inpatient stays that met denominator specifications for the Agency for Healthcare Research and Quality’s (AHRQ) medical mortality Inpatient Quality Indicators (IQIs).^21^ These IQIs measure in-hospital death for 6 medical conditions identified by principal diagnosis—acute myocardial infarction (AMI), heart failure, acute stroke, gastrointestinal hemorrhage, hip fracture, and pneumonia. We also included inpatient stays that met specifications for select AHRQ Patient Safety Indicators (PSIs) after elective operative procedures,^22^ which measure complications during and after surgery, including hemorrhage or hematoma, respiratory failure, pulmonary embolism or deep vein thrombosis, and sepsis. We limited the SID to 32 states for which a rural hospital merger occurred between 2009 and 2016. Data from the 2008 and 2017 to 2018 SID were included to ensure that each hospital had at least 1 premerger year and 2 postmerger years.

Discharge records were assigned to the merged or comparison group and were further categorized as occurring in the premerger or postmerger period, anchored by a merger index date (eAppendix 2 in the Supplement). For mergers, the premerger to postmerger period was determined from announcement and closure dates provided by the source or obtained through public information searches. For comparison hospitals, an index date corresponding to merger dates was randomly assigned within strata used for coarsened exact matching (CEM).

### Primary Independent and Dependent Variables

The primary exposure was the hospital’s merger status. The primary outcome was in-hospital deaths for IQIs and complications for PSIs. We examined the results overall for any IQI and for each of the individual measures. We additionally report the results overall for any PSI and for the individual PSI measures.

### Other Variables

We included all discharge-level variables specified in the IQI and PSI software in the models.^23,24^ Some exceptions were made if a variable could not be defined across all years owing to a change in coding. The IQI and PSI risk adjustments and all deviations from the specifications are provided in eAppendix 3 in the Supplement.

### Statistical Analysis

We used CEM to match discharges at merged and comparison hospitals by the hospital’s state, critical access status, bed number (<30 vs ≥30), and ownership (public, private nonprofit, or private for profit). Cells with merged hospitals but not for comparison hospitals were coarsened (ie, combined) with other cells to retain discharges at all merged hospitals. Discharges in cells with comparison hospitals but no merged hospitals were excluded to make the groups more comparable. Initially, we identified discharges at 172 merged hospitals and 549 comparison hospitals in rural areas. After CEM, the final sample consisted of discharges at 172 merged hospitals and 266 comparison hospitals, and imbalances between merged and comparison hospitals in the distributions of location (state), critical access status, bed number, and ownership were reduced (eTable 1 in the Supplement). Residual differences in these characteristics were adjusted for in regression models.

In descriptive analyses, we compared baseline characteristics of discharges at merged and comparison hospitals that met any IQI or PSI denominator specification. Owing to the large sample size of the SID, small differences can be statistically significant. Instead, we used standardized mean differences (SMDs) to assess balance in hospital and patient characteristics between merged and comparison groups.^25,26^ We also created trend graphs for the IQIs and PSIs 5 years before and after the merger or index date for merged and comparison hospitals.

Finally, we used linear probability models specifying mortality and complications as binary outcomes. We included a DID parameter, which was the interaction between merged (vs comparison hospitals) × postmerger year (vs premerger period). The DID coefficient can be interpreted as the difference in premerger to postmerger percentage point changes in rates of in-hospital mortality (or perioperative or postoperative complications) between merged and comparison hospitals. We present annual DID estimates for up to 5 years after the merger. The models produce robust SEs that account for within-hospital correlations and were conducted in Stata statistical software version 15.1 (StataCorp). We tested for statistical significance at the .01, .05 and .10 level using 2-sided tests, and significance was set at *P* < .05. Tests for the parallel trends assumption found no violations and are provided in eTable 2 in the Supplement. Data were analyzed from February to December 2020.

In sensitivity analyses, we used logistic regression (vs linear probability models) to assess the robustness of our results to model specification. We also measured outcomes for stays in the catchment areas of merged and comparison hospitals (vs stays treated at those hospitals) to examine associations between mergers and quality at the population level (eAppendix 4 in the Supplement).

## Results

### Study Sample

After CEM matching, our sample contained 303 747 IQI and 175 970 PSI discharges at 172 merged hospitals and 461 092 IQI and 278 070 PSI discharges at 266 comparison hospitals during the premerger period (Table 1). For each study population, baseline patient characteristics were comparable between merged and comparison hospitals in mean (SD) age (IQI: 72.9 [15.2] years vs 73.6 [15.0] years; PSI: 60.9 [15.5] years vs 61.9 [15.3] years), sex (IQI: 135 823 [44.7%] men and 167 924 [55.3%] women vs 211 443 [45.9%] men and 249 649 [54.1%] women; PSI: 64 855 [36.9%] men and 111 115 [63.1%] women vs 108 101 [38.9%] men and 169 969 [61.1%] women), and expected payer (IQI: 232 567 patients with Medicare [76.6%] vs 349 894 patients with Medicare [75.9%]; PSI: 88 607 patients with Medicare [50.4%] vs 141 613 patients with Medicare [50.9%]). Baseline characteristics were also similar between groups in community income, urban/rural location, number of chronic conditions, All Patient Refined Diagnosis Related Group risk-of-mortality score, select comorbidities, and travel distance (Table 1). Additional baseline comorbidities also were similar across the merged and comparison groups (eTable 3 in the Supplement). Patient baseline characteristics remained similar between groups in the postmerger period (eTable 4 in the Supplement).

**Table 1.** Baseline Patient Characteristics for Discharges in the Premerger Period at Study Hospitals After Coarsened Exact Matching^a^

Characteristic	Any IQI			Any PSI		
	Hospitals, No. (%)		SMD	Hospitals, No. (%)		SMD
	Merged (n = 303 747)	Comparison (n = 461 092)		Merged (n = 175 970)	Comparison (n = 278 070)	
Age, mean (SD), y	72.9 (15.2)	73.6 (15.0)	−0.04	60.9 (15.5)	61.9 (15.3)	−0.06
Sex						
Men	135 823 (44.7)	211 443 (45.9)	−0.02	64 855 (36.9)	108 101 (38.9)	−0.04
Women	167 924 (55.3)	249 649 (54.1)	0.02	111 115 (63.1)	169 969 (61.1)	0.04
Expected payer						
Medicare	232 567 (76.6)	349 894 (75.9)	0.02	88 607 (50.4)	141 613 (50.9)	−0.01
Medicaid	17 189 (5.7)	26 251 (5.7)	0.00	14 266 (8.1)	20 792 (7.5)	0.02
Private insurance	36 748 (12.1)	56 349 (12.2)	0.00	63 080 (35.8)	95 082 (34.2)	0.03
Self-pay or no charge	10 842 (3.6)	15 288 (3.3)	0.01	4184 (2.4)	7799 (2.8)	−0.03
Other	5789 (1.9)	8763 (1.9)	0.00	4890 (2.8)	11 181 (4.0)	−0.07
Community income, quartile						
First (lowest)	135 881 (44.7)	198 443 (43.0)	0.03	68 972 (39.2)	106 488 (38.3)	0.02
Second	110 933 (36.5)	157 690 (34.2)	0.05	70 911 (40.3)	99 477 (35.8)	0.09
Third	40 326 (13.3)	79 912 (17.3)	−0.11	26 121 (14.8)	52 832 (19.0)	−0.11
Fourth (highest)	8147 (2.7)	15 397 (3.3)	−0.04	5779 (3.3)	13 553 (4.9)	−0.08
Location of residence						
Metropolitan	59 526 (19.6)	78 392 (17.0)	0.07	36 101 (20.5)	43 944 (15.8)	0.12
Rural, metropolitan-adjacent	162 086 (53.4)	225 998 (49.0)	0.09	75 235 (42.8)	121 708 (43.8)	−0.02
Rural, remote	81 930 (27.0)	156 298 (33.9)	−0.15	64 517 (36.7)	112 206 (40.4)	−0.08
Chronic conditions, No.						
None	3090 (1.0)	6019 (1.3)	−0.03	7056 (4.0)	10 024 (3.6)	0.02
1	8413 (2.8)	14 499 (3.1)	−0.02	19 775 (11.2)	28 221 (10.1)	0.04
2	16 233 (5.3)	26 099 (5.7)	−0.01	25 162 (14.3)	38 209 (13.7)	0.02
≥3	276 011 (90.9)	414 475 (89.9)	0.03	123 977 (70.5)	201 616 (72.5)	−0.05
APR-DRG mortality risk score, mean (SD)	2.2 (0.9)	2.2 (0.8)	0.01	1.3 (0.6)	1.3 (0.6)	−0.02
Select comorbidities^b^						
Congestive heart failure	42 179 (13.9)	67 876 (14.7)	−0.02	6005 (3.4)	10 359 (3.7)	−0.02
Chronic pulmonary disease	111 349 (36.7)	171 419 (37.2)	−0.01	27 631 (15.7)	45 771 (16.5)	−0.02
Peripheral vascular disease	25 303 (8.3)	39 613 (8.6)	−0.01	8917 (5.1)	15 799 (5.7)	−0.03
Diabetes^c^	104 274 (34.3)	153 312 (33.2)	0.02	36 936 (21.0)	59 342 (21.3)	−0.01
Hypertension	196 733 (64.8)	294 600 (63.9)	0.02	95 283 (54.1)	154 636 (55.6)	−0.03
MSUD^d^	58 924 (19.4)	88 212 (19.1)	0.01	24 555 (14.0)	40 525 (14.6)	−0.02
Distance to hospital, mean (SD), mi	5.8 (6.1)	5.8 (6.2)	0.01	6.8 (6.8)	6.3 (6.4)	0.08

Abbreviations: APR-DRG, All Patient Refined Diagnosis Related Group; IQI, Inpatient Quality Indicator; MSUD, mental or substance use disorder; PSI, Patient Safety Indicator; SMD, standardized mean difference.

^a^ Data are taken from up to 10 baseline years, depending on the year of the merger or index date; comparison hospitals were randomly assigned an index date corresponding to the year of merged hospitals in the strata determined by the matching variables. The Agency for Healthcare Research and Quality’s Quality Indicator software was used to define the IQIs and PSIs (eAppendix 3 in the Supplement).

^b^ Complete list of comorbidities is provided in eTable 3 in the Supplement.

^c^ Includes diabetes with and without complications.

^d^ Includes alcohol abuse, depression, drug abuse, or psychoses.

### Volume Trends

**Figure 1.**
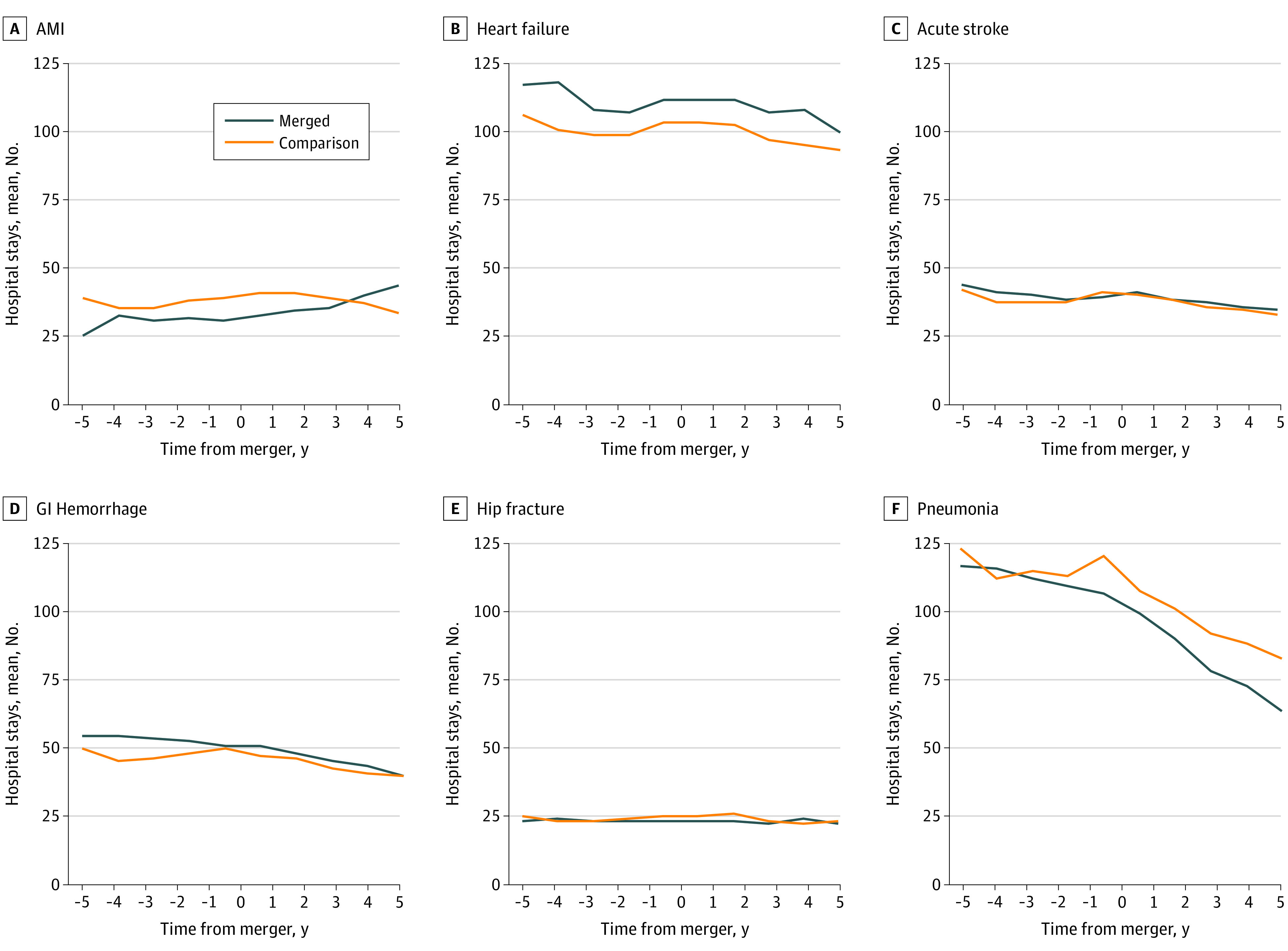
Mean Annual Number of Stays Across Hospitals for Each Inpatient Quality Indicator Before and After Mergers All hospitals were required to have at least 1 year of premerger and 2 years of postmerger data. The set of discharges at hospitals included in the 3-, 4-, and 5-year means before and after the merger is for a different set of hospitals than the full sample. AMI indicates acute myocardial infarction; and GI, gastrointestinal.

Figure 1 displays trends in the volume of inpatient stays for each of the 6 medical conditions in the premerger and postmerger periods (eTable 5 in the Supplement). From 4 years to 1 year premerger, the mean number of AMI stays at merged hospitals remained stable annually at approximately 24 to 26 stays (translating to approximately 1 admission every other week) but increased from a mean (SD) of 26 (62) stays per year at 1 year postmerger to 35 (86) stays per year at 5 years postmerger, or by 35%. For merged hospitals, the median (interquartile range) number of AMI stays was 7 (15) stays at 1 year postmerger and 5 (14) stays at 5 years postmerger; and for the 113 merged hospitals with 5 years of postmerger data, 41 had an increase in AMI stays. In contrast, the mean AMI volume declined after 2 years postmerger for comparison hospitals. We observed an inverse association between AMI mortality rates and volumes in the overall study sample (eFigure 2 in the Supplement). Mean volumes of stays for heart failure, stroke, gastrointestinal hemorrhage, and pneumonia decreased steadily over the period for both merged and comparison hospitals, but remained stable for hip fracture (eTable 5 in the Supplement). The mean volume of elective procedures also decreased similarly for both merged and comparison hospitals (eFigure 1 in the Supplement).

### Mortality and Complications

**Figure 2.**
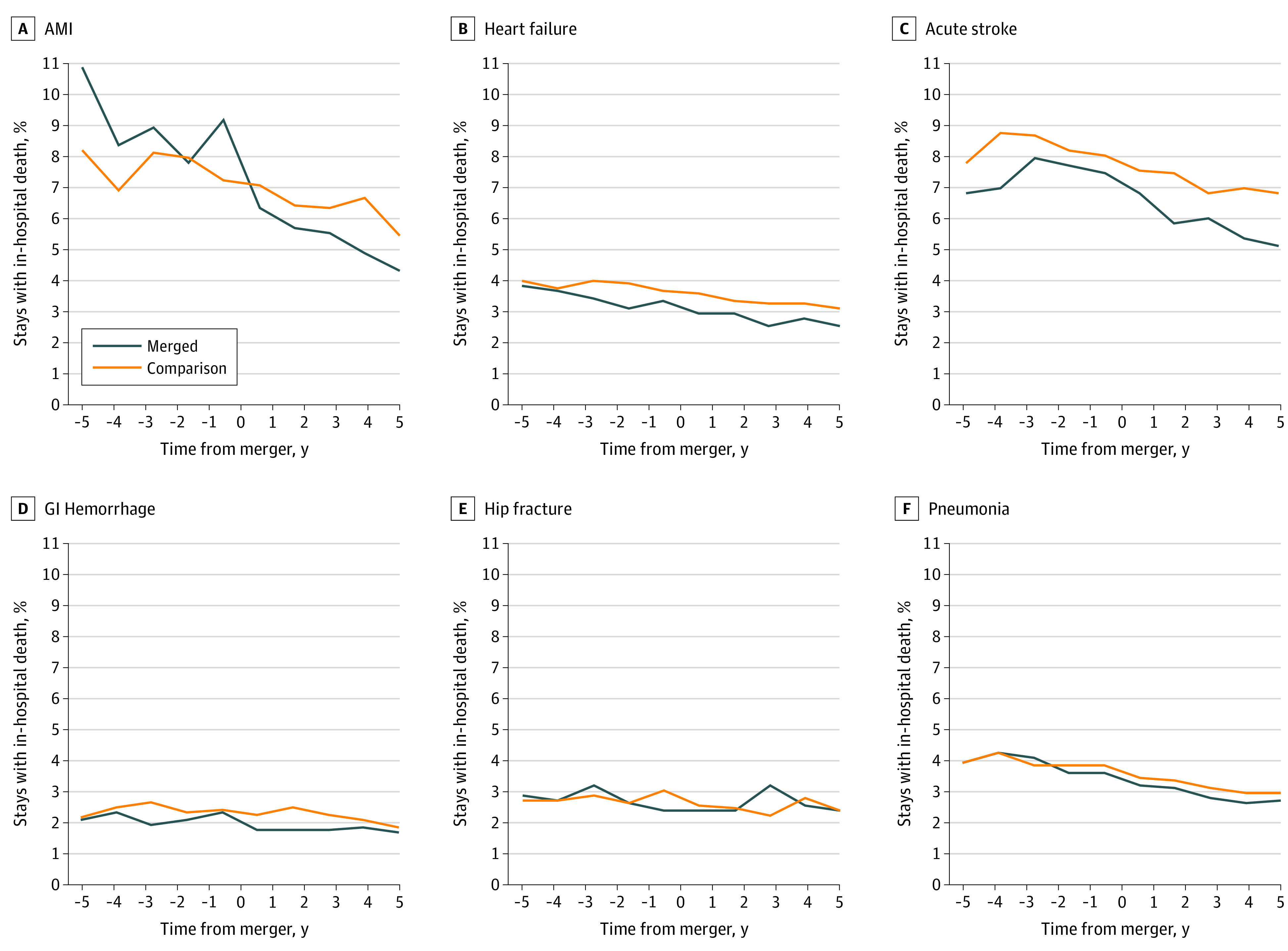
Percentage of All Stays That Resulted in In-Hospital Death for Each Inpatient Quality Indicator Before and After Mergers All hospitals were required to have at least 1 year of premerger and 2 years of postmerger data. The set of deaths included in the 3-, 4-, and 5-year percentages before and after the merger is for a different set of hospitals than the full sample. AMI indicates acute myocardial infarction; and GI, gastrointestinal.

Figure 2 displays trends in the in-hospital mortality rates for each of the 6 medical conditions before and after mergers (eTable 6 in the Supplement). The annual mortality rate for AMI stays fluctuated between 7.8% and 10.9% during the premerger period at merged hospitals but declined to 6.3% at 1 year postmerger and continued to decrease to 4.3% at 5 years postmerger. Mortality rates for heart failure, stroke, and pneumonia decreased steadily over the study period but remained stable for gastrointestinal hemorrhage and hip fracture for both merged and comparison hospitals (eTable 6 in the Supplement). Complications after elective procedures also decreased similarly for both merged and comparison hospitals.

### DID Results

Table 2 presents unadjusted premerger and postmerger mortality and complication rates for merged and comparison hospitals and adjusted DID estimates from the linear probability models. Consistent with the downward trends in Figure 2, the observed mortality rate decreased across all 6 conditions at both merged and comparison hospitals. Overall, the risk of mortality of all stays combined for 6 conditions had a greater decrease at merged hospitals than at comparison hospitals postmerger, from a difference of −0.443 (95% CI, −0.813 to 0.073) percentage points at 1 year (*P* = .02) to −0.757 (95% CI, −1.348 to −0.166) percentage points at 5 years (*P* = .01). Because mortality rates had decreased for both groups of hospitals (ie, negative premerger to postmerger change), a greater reduction at merged hospitals is represented by a negative estimate.

**Table 2.** Changes in Quality and Patient Safety for Stays at Hospitals That Merged vs at Comparison Hospitals

Quality indicator	In-hospital death (IQI) or complication (PSI), %^a^				Pre-post difference between merged and comparison hospitals, DID estimate % (95% CI)^b^				
	Merged hospitals		Comparison hospitals		Model 1		Model 2 (3 y postmerger)^c^	Model 3 (4 y postmerger)^c^	Model 4 (5 y postmerger)^c^
	Premerger	Postmerger	Premerger	Postmerger	1 y postmerger	2 y postmerger			
Any IQI mortality	4.3	3.2	4.4	3.8	−0.443 (−0.813 to 0.073)^d^	−0.476 (−0.881 to −0.072)^d^	−0.541 (−0.965 to −0.117)^d^	−0.656 (−1.181 to −0.132)^d^	−0.757 (−1.348 to −0.166)^d^
AMI	9.4	5.0	7.9	6.3	−1.755 (−2.825 to −0.685)^e^	−1.601 (−2.797 to −0.406)^e^	−1.615 (−2.98 to −0.25)^d^	−2.039 (−3.388 to −0.691)^e^	−1.095 (−2.572 to 0.382)
Heart failure	3.5	2.7	3.8	3.3	−0.491 (−1.004 to 0.023)^f^	−0.325 (−0.83 to 0.18)	−0.658 (−1.204 to −0.112)^d^	−0.634 (−1.28 to 0.012)^f^	−0.756 (−1.448 to −0.064)^d^
Acute stroke	7.5	5.8	8.2	7.2	−0.389 (−1.392 to 0.613)	−0.696 (−1.839 to 0.447)	−0.278 (−1.351 to 0.796)	−1.078 (−2.244 to 0.089)^f^	−1.667 (−3.05 to −0.283)^d^
GI hemorrhage	2.3	1.8	2.5	2.2	−0.409 (−0.884 to 0.067)^f^	−0.493 (−1.019 to 0.034)^f^	−0.295 (−0.859 to 0.269)	−0.039 (−0.637 to 0.559)	−0.124 (−0.714 to 0.467)
Hip fracture	2.8	2.4	2.9	2.4	0.054 (−0.567 to 0.674)	−0.01 (−0.622 to 0.602)	0.591 (−0.129 to 1.311)	−0.225 (−1.118 to 0.668)	−0.039 (−0.997 to 0.919)
Pneumonia	4.0	2.8	4.0	3.2	−0.233 (−0.71 to 0.245)	−0.356 (−0.88 to 0.168)	−0.552 (−1.163 to 0.059)^f^	−0.535 (−1.195 to 0.124)	−0.862 (−1.681 to −0.042)^d^
Any PSI complication	1.8	1.6	1.8	1.6	−0.084 (−0.346 to 0.177)	−0.122 (−0.406 to 0.162)	−0.006 (−0.29 to 0.277)	−0.216 (−0.538 to 0.106)	−0.115 (−0.445 to 0.214)

Abbreviations: AMI, acute myocardial infarction; DID, difference-in-differences; GI, gastrointestinal; IQI, Inpatient Quality Indicator; PSI, Patient Safety Indicator.

^a^ The premerger and postmerger period descriptive data are based on all premerger and postmerger years available from each hospital, up to 10 years before the merger and 10 years after the merger. Sample sizes in the premerger and postmerger periods are shown in eTable 10 in the Supplement.

^b^ DID estimates are from linear probability models and can be interpreted as the premerger postmerger-percentage point difference between merged and comparison hospitals in the percentage of admitted patients who died in the hospital (IQI) or who experienced complication (PSI). All models are adjusted according to the Agency for Healthcare Research and Quality’s Quality Indicator software, with some exceptions, plus additional patient, hospital, and community characteristics (eAppendix 3 in the Supplement). For both the merged and comparison groups, the rate of the IQIs and PSIs generally decreased from the premerger to the postmerger period. Thus, a negative DID estimate indicates that the decrease in the intervention group was greater than the decrease in the comparison group.

^c^ The third, fourth, and fifth postmerger year models are separate models that include only hospitals with 3 or more, 4 or more, and 5 or more postmerger years of data, respectively.

^d^ *P* < .05.

^e^ *P* < .01.

^f^ *P* < .10.

In-hospital mortality among AMI stays at merged hospitals decreased by 4.4 percentage points from the premerger to the postmerger period (from 9.4% to 5.0%) but decreased by less magnitude among AMI stays at comparison hospitals (1.6 percentage points, from 7.9% to 6.3%). This was confirmed with the adjusted DID estimates, which show that the difference in risk of AMI mortality 1 year postmerger was −1.755 (95% CI, −2.825 to −0.685) percentage points greater at merged hospitals than at comparison hospitals (*P* < .001). This trend continued up to 4 years postmerger (DID, −2.039 [95% CI, −3.388 to −0.691] percentage points; *P* < .01). The decrease in the risk of mortality was also greater for heart failure stays at merged vs comparison hospitals at 3 and 5 years postmerger (DID, −0.756 [95% CI, −1.448 to −0.064] percentage points; *P* = .03), as well as for acute stroke stays (DID, −1.667 [95% CI, −3.050 to −0.283] percentage points; *P* = .02) and pneumonia stays (DID, −0.862 [95% CI, −1.681 to −0.042] percentage points; *P* = .04) at 5 years postmerger (Table 2).

No significant difference was found between merged and comparison hospitals in changes of complication rates after elective procedures for the PSIs overall or for the individual PSIs (eTable 7 and eFigure 1 in the Supplement). The estimates from the logistic regression models were robust with respect to the direction and significance of the linear probability estimates (eTable 8 in the Supplement). Except for heart failure, the catchment area results were null (eTable 9 in the Supplement).

## Discussion

In this case-control study, we found a significantly greater reduction in inpatient mortality for several common conditions (ie, AMI, heart failure, acute stroke, and pneumonia) among patients admitted to rural hospitals that merged or were acquired than among patients admitted to rural hospitals that remained independent. Although mortality for these conditions declined for all hospitals during the study period, the premerger to postmerger reductions at merged hospitals exceeded those at comparison hospitals after adjusting for patient, hospital, and community characteristics. Understanding the impact of mergers and acquisitions on rural hospital quality is critical for informing rural health policy and health care management. These findings suggest that rural hospital mergers were associated with quality improvement.

We found significantly greater improvement in mortality for patients with AMI at merged hospitals than comparison hospitals for the first 4 years after mergers. This could be because merged hospitals had more resources and support to adopt defined clinical pathways available for AMI through transfer of technology and expertise from the larger system. Previous research has found that adoption of hospital strategies for AMI management is associated with lower 30-day risk-standardized mortality rates,^27^ although it is not clear how quickly improvement can be achieved.^28^ It is also worth noting that the mean volume of AMI stays increased at merged hospitals after mergers. The inverse association between AMI mortality rates and inpatient volumes has been well documented in the literature,^29,30,31,32^ and was also observed in this study, but volume-outcome research thus far has not been specific to rural hospitals.^33^

Significant improvements in mortality for the other 3 conditions (ie, heart failure, stroke, and pneumonia) did not occur immediately after mergers but rather 3 to 5 years later. This timeframe is consistent with research indicating that adoption of quality improvement approaches is complex and requires internal diffusion within given health care organizations prior to improved outcomes.^34,35^ Merged hospitals achieved greater improvement in mortality outcomes for stays with these conditions than comparison hospitals did. Heart failure and pneumonia are high-volume conditions in rural areas with aging populations, and ensuring timely initial evaluation and treatment for acute stroke is particularly challenging. With reduced access to care, rural residents with these conditions are at greater risk of death than their urban counterparts.^36,37^ Mergers can allow partnerships with urban hospitals to facilitate implementation of clinical pathways and protocols for improving patient outcomes.^38^ Through mergers, rural hospitals can also gain access to capital investment in electronic health records and clinical decision support systems for enhancing technological capabilities.^39^ Furthermore, sharing staff and expertise as part of the merger can help alleviate workforce shortages and improve the hospital’s clinical services.

The findings of this study regarding the positive outcomes associated with mergers in rural hospital quality challenge a common argument in prior research that hospital consolidation is likely to result in greater market power and higher prices but poorer quality. The association between mergers and quality of care appears to function differently in urban vs rural settings. In urban markets, hospital consolidation was found to either hurt quality^12,13,15^ or have no measurable impact.^11,16^ In contrast, although a study by O’Hanlon and colleagues^17^ reported no differences in patient experience and readmissions for rural hospitals with system affiliation using self-reported hospital data, our study found that mergers were associated with better patient outcomes in rural hospitals using all-payer discharge-level data and indicators of hospital quality.^40^ Future research should examine whether there are differential outcomes for rural hospitals that are acquired by large hospital systems than for rural hospitals that merge locally with another hospital, or for those that were already merged or affiliated before a second merger.

### Limitations

This study has several limitations. First, although our methods adjusted for patient, hospital, and community characteristics through use of CEM and DID models, there are potentially unmeasured variables endogenous to the likelihood of merger and the quality indicators. Some imbalances across hospitals persisted after matching, but we controlled for these variables, and discharges in the merged and comparison groups were similar on all factors examined. Second, our study spans the *International Classification of Diseases, Ninth Revision, Clinical Modification* (*ICD-9-CM*) and the *ICD-10-CM* periods, affecting use of the quality indicators. Third, if rural hospitals that merge significantly curtail service lines or transfer patients with higher risk to urban centers, or if patients die during transfer to another hospital or out of the hospital, this could account for a greater reduction in mortality for these hospitals. However, we did not find evidence of accelerating decreases in volume after mergers in the clinical conditions examined.

## Conclusions

This case-control study found that rural hospital mergers were associated with decreased in-hospital mortality for AMI and several other conditions. These findings indicate that mergers of rural hospitals are not necessarily associated with adverse changes in the quality of care at these hospitals. Mergers may enable rural hospitals to improve quality of care through access to needed financial, clinical, and technological resources, which is important to enhancing rural health and reducing urban-rural disparities in quality. This hypothesis needs to be assessed using data sources that capture data both on quality and hospital resources. Despite the positive association between mergers and quality, merging may not be an option for some rural hospitals, which may remain financially vulnerable and thus at risk of eliminating services or shuttering.

## Notes

Supplement. **eAppendix 1.** Merger Definition **eAppendix 2.** Assignment of Merger Dates Across Data Sources **eAppendix 3.** Summary of Deviations From Quality Indicator Software **eAppendix 4.** Catchment Area Sensitivity Analysis **eTable 1.** Baseline Characteristics of Study Hospitals Before and After Coarsened Exact Matching **eTable 2.** Tests for Premerger Period Parallel Trends Between the Merged and Comparison Groups **eTable 3.** Baseline Comorbidities Among Stays for Time-Sensitive Conditions and Elective Procedures for the Merged and Comparison Groups **eTable 4.** Comparison of Patient Mix in the Premerger and Postmerger Periods **eTable 5.** Mean Hospital-Level Number of Stays for Each IQI Before and After Merger **eTable 6.** In-Hospital Mortality Among Stays for Each IQI Before and After Merger **eTable 7.** Changes in the Individual PSIs for Stays at Hospitals That Merged and at Comparison Hospitals **eTable 8.** Changes in Quality and Patient Safety for Stays at Hospitals That Merged and at Comparison Hospitals Assessed Using Logistic Regression Models **eTable 9.** Changes in Quality and Patient Safety for Stays in the Catchment Areas of Hospitals That Merged and in the Catchment Areas of Comparison Hospitals **eTable 10.** Premerger and Postmerger Period Sample Sizes **eFigure 1.** Mean Annual Number of Stays for Elective Procedures Across Hospitals and Percentage of All Stays With Any PSI Complication **eFigure 2.** Hospital Scatter Plots of Inpatient Volume and In-Hospital Mortality Rates for the IQIs
