# Supplementary material for: Quality of Care Before and After Mergers and Acquisitions of Rural Hospitals
Source: JAMA Netw Open. 2021 Sep 20;4(9):e2124662. doi: 10.1001/jamanetworkopen.2021.24662 (PMC8453322; doi:10.1001/jamanetworkopen.2021.24662)
Supplement: Supplement. — eAppendix 1. Merger Definition eAppendix 2. Assignment of Merger Dates Across Data Sources eAppendix 3. Summary of Deviations From Quality Indicator Software eAppendix 4. Catchment Area Sensitivity Analysis eTable 1. Baseline Characteristics of Study Hospitals Before and After Coarsened Exact Matching eTable 2. Tests for Premerger Period Parallel Trends Between the Merged and Comparison Groups eTable 3. Baseline Comorbidities Among Stays for Time-Sensitive Conditions and Elective Procedures for the Merged and Comparison Groups eTable 4. Comparison of Patient Mix in the Premerger and Postmerger Periods eTable 5. Mean Hospital-Level Number of Stays for Each IQI Before and After Merger eTable 6. In-Hospital Mortality Among Stays for Each IQI Before and After Merger eTable 7. Changes in the Individual PSIs for Stays at Hospitals That Merged and at Comparison Hospitals eTable 8. Changes in Quality and Patient Safety for Stays at Hospitals That Merged and at Comparison Hospitals Assessed Using Logistic Regression Models eTable 9. Changes in Quality and Patient Safety for Stays in the Catchment Areas of Hospitals That Merged and in the Catchment Areas of Comparison Hospitals eTable 10. Premerger and Postmerger Period Sample Sizes eFigure 1. Mean Annual Number of Stays for Elective Procedures Across Hospitals and Percentage of All Stays With Any PSI Complication eFigure 2. Hospital Scatter Plots of Inpatient Volume and In-Hospital Mortality Rates for the IQIs [file jamanetwopen-e2124662-s001.pdf]

Supplemental Online Content

Jiang HJ, Fingar KR, Liang L, Henke RM, Gibson TP. Quality of care before and after mergers and acquisitions of rural hospitals. *JAMA Netw Open*. 2021;4(9):e2124662. doi:10.1001/jamanetworkopen.2021.24662

eAppendix 1. Merger Definition
eAppendix 2. Assignment of Merger Dates Across Data Sources
eAppendix 3. Summary of Deviations From Quality Indicator Software
eAppendix 4. Catchment Area Sensitivity Analysis
eTable 1. Baseline Characteristics of Study Hospitals Before and After Coarsened Exact Matching
eTable 2. Tests for Premerger Period Parallel Trends Between the Merged and Comparison Groups
eTable 3. Baseline Comorbidities Among Stays for Time-Sensitive Conditions and Elective Procedures for the Merged and Comparison Groups
eTable 4. Comparison of Patient Mix in the Premerger and Postmerger Periods
eTable 5. Mean Hospital-Level Number of Stays for Each IQI Before and After Merger
eTable 6. In-Hospital Mortality Among Stays for Each IQI Before and After Merger
eTable 7. Changes in the Individual PSIs for Stays at Hospitals That Merged and at Comparison Hospitals
eTable 8. Changes in Quality and Patient Safety for Stays at Hospitals That Merged and at Comparison Hospitals Assessed Using Logistic Regression Models
eTable 9. Changes in Quality and Patient Safety for Stays in the Catchment Areas of Hospitals That Merged and in the Catchment Areas of Comparison Hospitals
eTable 10. Premerger and Postmerger Period Sample Sizes
eFigure 1. Mean Annual Number of Stays for Elective Procedures Across Hospitals and Percentage of All Stays With Any PSI Complication
eFigure 2. Hospital Scatter Plots of Inpatient Volume and In-Hospital Mortality Rates for the IQIs

This supplemental material has been provided by the authors to give readers additional information about their work.

© 2021 Jiang HJ et al. *JAMA Network Open.*

eAppendix 1. Merger Definition

We identified hospital mergers and acquisitions between 2009 and 2016 using data from Irving Levin Associates and supplemented this list with data from the American Hospital Association's (AHA) Annual Survey for hospitals that reported a change in system membership and for which we confirmed it was a merger or acquisition through public information searches. Together, these sources represent mergers, acquisitions, and changes in hospital affiliation, which we refer to as mergers. Hospital mergers may include hospitals that were already affiliated with another health system or that had been previously acquired and were being sold again. Hospitals that were a part of an affiliation between two multi-hospital systems were not included in the definition of mergers because these hospitals were not individually identified in the Levin data.

© 2021 Jiang HJ et al. *JAMA Network Open.*

eAppendix 2. Assignment of Merger Dates Across Data Sources

The determination of pre-post periods varied by data source. For mergers in Levin, the time before the announcement date was defined as the premerger period and the time after the transaction closure date was defined as the postmerger period. For those mergers reported in the American Hospital Association Annual Survey but not in Levin (only 29 out of 172 merged hospitals), we found announcement and transaction dates through web searches for 21 of these hospitals but set the announcement and transaction dates to January and December for the remaining hospitals. Discharges were excluded if they occurred between the announcement and transaction closure dates.

© 2021 Jiang HJ et al. *JAMA Network Open.*

eAppendix 3. Summary of Deviations From Quality Indicator Software

Whereas the Patient Safety Indicator (PSI) specifications restrict PSI 11 (postoperative respiratory failure) and PSI 13 (postoperative sepsis) to elective discharges, the specifications for PSI 09 (perioperative hemorrhage or hematoma) and PSI 12 (perioperative pulmonary embolism/deep venous thrombosis) do not. For the purposes of this analysis, PSI 09 and PSI 12 also are limited to elective discharges. Additionally, the following modifications to the Inpatient Quality Indicator (IQI) and PSI software were made to accommodate the inclusion of more years of data or data from more states.

- The IQI models did not adjust for the following All Patient Refined Diagnosis Related Groups (APR-DRGs) because they did not exist in version 25 of the APR-DRG software, which was used by version 6 of the IQI software:
    - ADX009: Extracorporeal membrane oxygenation (ECMO)
    - ADX181: Lower extremity arterial procedures
    - ADX182: Other peripheral vascular procedures
- The PSI models did not adjust for the following Modified Diagnosis-Related Groups (MDRGs) because they did not exist in version 25 of the DRG software, which was used by version 6.02 of the PSI software:
    - MDRG_540: Endovascular cardiac valve replacement
    - MDRG_541: Aortic and heart assist procedures except pulsation balloon
    - MDRG_542: Other major cardiovascular procedures
    - MDRG_543: Percutaneous intracardiac procedures
    - MDRG_815: Back and neck procedures except spinal fusion or disc device/neurostimulator
    - MDRG_7705: Allogenic or autologous bone marrow transplant
- The PSI specifications account for whether a diagnosis was present on admission and the day of the stay on which the procedure occurred.
    - Present on admission was not considered in our analysis because multiple states do not send this information to the Healthcare Cost and Utilization Project.
    - The day of the procedure was not available for Oklahoma and West Virginia in any year and was not available for Illinois or Washington in 2008 or for Ohio from 2008 to 2011. For these states, the day of procedure was not considered, but it was considered for all other states.

Note that during the International Classification of Diseases, Ninth Revision, Clinical Modification (ICD-9-CM) period, we used version 6.0 of the IQI software and version 6.02 of the PSI software. During the ICD-10-CM period, we used version 2020 of the IQI and PSI software.

Risk-adjusters for the IQIs include sex and age, Major Diagnostic Categories (MDCs), 3M All Patient Refined Diagnosis Related Groups (APR-DRGs) and Risk of Mortality subclass categories, transfers into the hospital, and other indicator-specific risk stratifiers. For the PSIs, risk-adjusters included sex and age, MDCs, Modified Medicare Severity-Diagnosis Related Group categories, comorbidities, transfers into the hospital, and other indicator-specific risk stratifiers.

In addition to the deviations described above, our models included additional patient, hospital, and community variables. Patient variables were primary expected payer, location of residence defined by Rural-Urban Continuum Codes,[1] and number of chronic conditions.[2] Hospital variables included bed size, critical access status, ownership, full-time equivalent registered nurses adjusted for inpatient days, and resident-to-bed ratio. Community variables corresponding to the patient's ZIP Code of residence included median income quartile, percent insured, percent unemployed, percent with a bachelor's degree or higher, percent female, and mean age. Finally, community variables corresponding to the patient's county of residence included number of Federally Qualified Health Centers and primary care physicians per capita. Data were missing for the ZIP Code-level variables for 2% of discharges, for expected payer for 0.4% of discharges, and for location of residence for 0.1% of discharges, which were excluded from regression analyses.

---

[1] US Department of Agriculture. Rural-Urban Continuum Codes. Last updated December 10, 2020. Accessed March 17, 2021. https://www.ers.usda.gov/data-products/rural-urban-continuum-codes.aspx

[2] Healthcare Cost and Utilization Project. Chronic Condition Indicator (CCI) for ICD-10-CM (beta version). Agency for Healthcare Research and Quality; last modified November 12, 2020. Accessed March 17, 2021. https://www.hcup-us.ahrq.gov/toolssoftware/chronic_icd10/chronic_icd10.jsp

eAppendix 4. Methods, Catchment Area Sensitivity Analysis

To define hospital catchment areas, we assembled all discharges by residential ZIP Code for each hospital and ranked ZIP Codes in descending order. The collection of ZIP Codes that accounted for up to 75% of a hospital's total discharges was defined as the hospital's catchment area. If a ZIP Code fell in both the catchment area of a merged hospital and a comparison hospital, it was counted in the catchment area of the former. Otherwise, if a ZIP Code fell in the catchment areas of two hospitals, it was assigned to the hospital for which it accounted for the greatest percentage of the hospital's discharges.

eTable 1. Baseline Characteristics of Study Hospitals Before and After Coarsened Exact Matching

	**Before matching**			**After matching**		
**Characteristic**	**Merged hospitals**	**Initial comparison hospitals**	**SMD**	**Merged hospitals**	**Final comparison hospitals**	**SMD**
**Total hospitals, N**	**172**	**549**	**N/A**	**172**	**266**	**N/A**
**Matching variables**						
Critical access hospital, %	34.3	58.8	-0.51	34.3	45.1	-0.25
Bed size, mean	65.9	49.8	0.31	65.9	52.4	-0.07
Bed size, 30+, %	64.0	43.4	0.42	64.0	56.4	0.14
Ownership, %						
Public	19.8	54.3	-0.77	19.8	33.5	-0.38
Private nonprofit	64.0	44.8	0.39	64.0	65.4	-0.03
Private for profit	16.3	0.9	0.57	16.3	1.1	0.38

Abbreviations: N/A, not applicable; SMD, standardized mean difference.

Note: Data come from the year before the merger/index date; comparison hospitals were randomly assigned an index date corresponding to the year of merged hospitals in the strata determined by the matching variables. SMDs are considered small if less than 0.20, medium if less than 0.50, large if greater than 0.80.
Source: Agency for Healthcare Research and Quality, Healthcare Cost and Utilization Project, State Inpatient Databases, 2008–2018.

© 2021 Jiang HJ et al. *JAMA Network Open.*

eTable 2. Tests for Premerger Period Parallel Trends Between the Merged and Comparison Groups

Outcome	Interaction of linear premerger trend*intervention	
	DID	*P*-value
**Any IQI, stays at the hospital**	-0.001	0.178
AMI mortality	0.000	0.790
Heart failure mortality	-0.001	0.185
Acute stroke mortality	0.000	0.851
GI hemorrhage mortality	-0.001	0.123
Hip fracture mortality	0.000	0.897
Pneumonia mortality	-0.001	0.167
**Any PSI, stays at the hospital**	0.000	0.239
Perioperative hemorrhage or hematoma	0.000	0.978
Postoperative respiratory failure	0.000	0.504
Perioperative PE/DVT	0.000	0.554
Postoperative sepsis	0.000	0.213

Abbreviations: AMI, acute myocardial infarction; DID, difference-in-differences; DVT, deep vein thrombosis; GI, gastrointestinal; IQI, Inpatient Quality Indicator; PE, pulmonary embolism; PSI, Patient Safety Indicator.

Note: The parallel trends assumption was tested by interacting a continuous variable for time before the merger with a dichotomous variable for the intervention group and limiting the data set to discharges in the pre period.

Source: Agency for Healthcare Research and Quality, Healthcare Cost and Utilization Project, State Inpatient Databases, 2008–2018.

eTable 3. Baseline Comorbidities Among Stays for Time-Sensitive Conditions and Elective Procedures for the Merged and Comparison Groups

Characteristic	Discharges in the premerger period, any IQI			Discharges in the premerger period, any PSI		
	Merged hospitals	Comparison hospitals	SMD	Merged hospitals	Comparison hospitals	SMD
**Total discharges, pre-period, N**	**303,747**	**461,092**	**N/A**	**175,970**	**278,070**	**N/A**
Comorbidity, %						
Alcohol abuse	3.1	2.7	0.02	1.1	1.2	-0.01
Deficiency anemias	23.1	23.0	0.00	11.5	11.4	0.00
Rheumatoid arthritis/collagen vascular disease	3.1	3.3	-0.01	2.6	2.9	-0.02
Chronic blood loss anemia	3.1	2.8	0.02	1.5	1.4	0.01
Congestive heart failure	13.9	14.7	-0.02	3.4	3.7	-0.02
Chronic pulmonary disease	36.7	37.2	-0.01	15.7	16.5	-0.02
Coagulopathy	3.8	3.7	0.01	1.5	1.4	0.01
Depression	11.8	11.9	0.00	10.5	10.9	-0.01
Diabetes without chronic complications	28.8	28.0	0.02	18.2	18.9	-0.02
Diabetes w/chronic complications	5.5	5.3	0.01	2.8	2.5	0.02
Drug abuse	1.2	1.1	0.01	0.4	0.5	-0.02
Hypertension	64.8	63.9	0.02	54.1	55.6	-0.03
Hypothyroidism	15.1	15.4	-0.01	10.9	11.1	-0.01
Liver disease	2.0	2.0	0.00	1.0	0.9	0.01
Lymphoma	0.8	0.9	-0.01	0.3	0.3	0.00
Fluid and electrolyte disorders	28.3	27.9	0.01	8.2	8.5	-0.01
Metastatic cancer	1.7	1.9	-0.02	1.7	1.7	0.00
Other neurological disorders	10.2	10.1	0.00	4.2	4.3	0.00
Obesity	9.8	9.6	0.01	12.7	14.0	-0.04
Paralysis	2.4	2.1	0.02	1.0	0.9	0.01
Peripheral vascular disease	8.3	8.6	-0.01	5.1	5.7	-0.03
Psychoses	3.3	3.4	-0.01	2.0	2.0	0.00
Pulmonary circulation disease	2.4	2.3	0.00	0.8	0.8	0.00
Renal failure	20.5	20.5	0.00	4.8	4.9	0.00
Solid tumor without metastasis	2.5	2.7	-0.01	1.1	1.2	-0.01
Peptic ulcer disease with bleeding	0.0	0.0	0.00	0.0	0.0	0.00
Valvular disease	4.4	4.9	-0.02	2.6	2.6	0.00
Weight loss	4.4	4.7	-0.01	1.6	1.6	0.00

Abbreviations: IQI, Inpatient Quality Indicator; N/A, not applicable; PSI, Patient Safety Indicator; SMD, standardized mean difference.

Note: Data come from up to 10 baseline years, depending on the year of the merger/index date; comparison hospitals were randomly assigned an index date corresponding to the year of merged hospitals in the strata determined by the matching variables.

Source: Agency for Healthcare Research and Quality, Healthcare Cost and Utilization Project, State Inpatient Databases, 2008–2018.

© 2021 Jiang HJ et al. *JAMA Network Open.*

eTable 4. Comparison of Patient Mix in the Premerger and Postmerger Periods

Characteristic	Any IQI						Any PSI					
	Merged hospitals			Comparison hospitals			Merged hospitals			Comparison hospitals		
	Pre period	Post period	SMD	Pre period	Post period	SMD	Pre period	Post period	SMD	Pre period	Post period	SMD
**Total, N**	**303,747**	**269,881**	**N/A**	**461,092**	**444,902**	**N/A**	**175,970**	**151,484**	**N/A**	**278,070**	**259,891**	**N/A**
Age, years, mean	72.9	72.9	0.00	73.6	73.5	0.01	60.9	61.8	-0.06	61.9	62.5	-0.04
Males, %	44.7	45.7	-0.02	45.9	46.3	-0.01	36.9	38.5	-0.03	38.9	40.3	-0.03
Expected payer, %												
Medicare	76.6	76.1	0.01	75.9	76.5	-0.01	50.4	51.7	-0.03	50.9	52.7	-0.04
Medicaid	5.7	6.0	-0.01	5.7	6.0	-0.01	8.1	9.0	-0.03	7.5	8.3	-0.03
Private insurance	12.1	12.2	0.00	12.2	12.0	0.01	35.8	33.7	0.04	34.2	32.6	0.03
Self-pay/No charge	3.6	3.3	0.02	3.3	3.0	0.02	2.4	2.0	0.03	2.8	2.3	0.03
Other	1.9	2.3	-0.03	1.9	2.0	-0.01	2.8	3.2	-0.02	4.0	3.7	0.02
Community income, %												
Quartile 1 (lowest)	44.7	43.6	0.02	43.0	42.2	0.02	39.2	38.8	0.01	38.3	38.6	-0.01
Quartile 2	36.5	36.7	0.00	34.2	35.8	-0.03	40.3	39.9	0.01	35.8	37.0	-0.02
Quartile 3	13.3	14.5	-0.04	17.3	16.6	0.02	14.8	15.7	-0.02	19.0	18.0	0.03
Quartile 4 (highest)	2.7	2.6	0.00	3.3	3.3	0.00	3.3	3.2	0.00	4.9	4.4	0.02
Location of residence, %												
Metro	19.6	19.4	0.01	17.0	16.4	0.02	20.5	20.1	0.01	15.8	16.3	-0.01
Rural, metro adjacent	53.4	50.7	0.05	49.0	48.8	0.00	42.8	40.2	0.05	43.8	42.8	0.02
Rural, remote	27.0	29.9	-0.06	33.9	34.8	-0.02	36.7	39.6	-0.06	40.4	40.8	-0.01
Chronic conditions, %												
None	1.0	0.8	0.02	1.3	1.1	0.01	4.0	3.2	0.04	3.6	3.0	0.04
1	2.8	2.3	0.03	3.1	2.9	0.01	11.2	9.7	0.05	10.1	9.2	0.03
2	5.3	4.5	0.04	5.7	5.2	0.02	14.3	12.6	0.05	13.7	12.6	0.03
3+	90.9	92.4	-0.05	89.9	90.8	-0.03	70.5	74.5	-0.09	72.5	75.2	-0.06
APR-DRG mortality risk, mean	2.2	2.2	-0.05	2.2	2.2	-0.01	1.3	1.3	-0.07	1.3	1.4	-0.08
Select comorbidities, %												
Congestive heart failure	13.9	13.5	0.01	14.7	14.3	0.01	3.4	3.7	-0.02	3.7	3.9	-0.01
Chronic pulmonary disease	36.7	36.9	-0.01	37.2	37.0	0.00	15.7	16.9	-0.03	16.5	17.4	-0.02
Peripheral vascular disease	8.3	8.7	-0.01	8.6	8.4	0.01	5.1	5.5	-0.02	5.7	5.7	0.00
Diabetes[a]	34.3	35.6	-0.03	33.2	34.5	-0.03	21.0	22.2	-0.03	21.3	22.5	-0.03
Hypertension	64.8	64.0	0.02	63.9	62.8	0.02	54.1	56.9	-0.06	55.6	58.1	-0.05

© 2021 Jiang HJ et al. *JAMA Network Open.*

MSUD[b]	19.4	20.1	-0.02	19.1	19.2	0.00	14.0	16.0	-0.05	14.6	15.7	-0.03
Distance to hospital, mean	5.8	6.1	-0.04	5.8	6.0	-0.04	6.8	6.9	-0.02	6.3	6.4	-0.01

Abbreviations: APR-DRG, All Patient Refined Diagnosis Related Group; IQI, Inpatient Quality Indicator; MSUD, mental and/or substance use disorder; N/A, not applicable; PSI, Patient Safety Indicator; SD, standard deviation; SMD, standardized mean difference.

Note: Data come from up to 10 baseline years, depending on the year of the merger/index date; comparison hospitals were randomly assigned an index date corresponding to the year of merged hospitals in the strata determined by the matching variables. The Agency for Healthcare Research and Quality's Quality Indicator software was used to define the IQIs and PSIs (see Supplement, eMethods 2).

[a] Includes diabetes with and without complications.

[b] Includes alcohol abuse, depression, drug abuse, or psychoses.

Source: Agency for Healthcare Research and Quality, Healthcare Cost and Utilization Project, State Inpatient Databases, 2008–2018.

© 2021 Jiang HJ et al. *JAMA Network Open.*

eTable 5. Mean Hospital-Level Number of Stays for Each IQI Before and After Merger

	Premerger period, years premerger					Postmerger period, years postmerger				
**IQI**	**-5**	**-4**	**-3**	**-2**	**-1**	**1**	**2**	**3**	**4**	**5**
**Merged hospitals**										
**Any IQI stay**	333	338	323	318	318	313	301	283	281	260
AMI	20	26	24	25	24	26	27	28	32	35
Heart failure	93	94	86	85	89	89	89	86	86	79
Acute stroke	35	33	32	31	32	33	31	30	29	28
GI hemorrhage	44	44	43	43	41	41	39	37	35	32
Hip fracture	23	25	24	24	24	24	24	23	24	22
Pneumonia	117	116	113	110	107	100	91	79	74	64
**Comparison hospitals**										
**Any IQI stay**	339	312	314	316	334	319	311	288	279	267
AMI	31	28	28	30	31	33	33	31	29	26
Heart failure	84	80	79	79	83	83	82	77	76	74
Acute stroke	34	30	30	30	33	32	31	29	28	27
GI hemorrhage	41	37	38	39	40	38	38	35	33	32
Hip fracture	25	24	24	24	26	25	26	24	23	24
Pneumonia	124	113	115	114	121	108	102	93	89	83

Abbreviations: AMI, acute myocardial infarction; GI, gastrointestinal; IQI, Inpatient Quality Indicator.

Source: Agency for Healthcare Research and Quality, Healthcare Cost and Utilization Project, State Inpatient Databases, 2008–2018.

© 2021 Jiang HJ et al. *JAMA Network Open.*

eTable 6. In-Hospital Mortality Among Stays for Each IQI Before and After Merger

IQI	Premerger period, years premerger					Postmerger period, years postmerger				
	**-5**	**-4**	**-3**	**-2**	**-1**	**1**	**2**	**3**	**4**	**5**
**Merged hospitals**										
AMI	10.9	8.4	8.9	7.8	9.2	6.3	5.7	5.5	4.9	4.3
Heart failure	3.9	3.7	3.5	3.1	3.3	2.9	2.9	2.5	2.8	2.5
Acute stroke	6.9	7.0	8.0	7.7	7.5	6.8	5.8	6.0	5.3	5.1
GI hemorrhage	2.2	2.4	1.9	2.1	2.3	1.8	1.8	1.8	1.9	1.7
Hip fracture	2.9	2.7	3.2	2.6	2.4	2.4	2.4	3.2	2.6	2.4
Pneumonia	3.9	4.2	4.1	3.6	3.6	3.2	3.1	2.8	2.6	2.7
**Comparison hospitals**										
AMI	8.2	6.9	8.1	8.0	7.3	7.1	6.5	6.3	6.6	5.5
Heart failure	4.0	3.8	4.0	3.9	3.7	3.6	3.4	3.3	3.3	3.1
Acute stroke	7.8	8.8	8.7	8.2	8.0	7.6	7.4	6.9	7.0	6.8
GI hemorrhage	2.2	2.5	2.7	2.4	2.5	2.3	2.5	2.3	2.1	1.9
Hip fracture	2.7	2.7	2.9	2.6	3.1	2.5	2.5	2.2	2.8	2.3
Pneumonia	3.9	4.2	3.8	3.8	3.8	3.5	3.3	3.1	2.9	3.0

Abbreviations: AMI, acute myocardial infarction; GI, gastrointestinal; IQI, Inpatient Quality Indicator.

Source: Agency for Healthcare Research and Quality, Healthcare Cost and Utilization Project, State Inpatient Databases, 2008–2018.

© 2021 Jiang HJ et al. *JAMA Network Open.*

eTable 7. Changes in the Individual PSIs for Stays at Hospitals That Merged and at Comparison Hospitals

Quality indicator	% with complication				Pre-post difference, by year, between merged and comparison hospitals DID estimate (95% CI) in percentage points				
	Merged hospitals		Comparison hospitals		Model 1		Model 2[b]	Model 3[b]	Model 4[b]
	Pre period[a]	Post period[a]	Pre period[a]	Post period[a]	1 year post	2 years post	3 years post	4 years post	5 years post
**Any PSI complication**	1.8	1.6	1.8	1.6	-0.084 (-0.346, 0.177)	-0.122 (-0.406, 0.162)	-0.006 (-0.29, 0.277)	-0.216 (-0.538, 0.106)	-0.115 (-0.445, 0.214)
Perioperative hemorrhage or hematoma	0.5	0.4	0.5	0.3	0.008 (-0.112, 0.128)	0.038 (-0.061, 0.137)	0.05 (-0.061, 0.161)	0.011 (-0.124, 0.147)	0.006 (-0.113, 0.126)
Postoperative respiratory failure	0.8	0.7	0.8	0.7	-0.077 (-0.271, 0.117)	-0.034 (-0.226, 0.157)	0.018 (-0.181, 0.217)	-0.127 (-0.341, 0.087)	-0.247[c] (-0.496, 0.001)
Perioperative PE/DVT	0.4	0.3	0.4	0.3	0.000 (-0.095, 0.095)	-0.004 (-0.105, 0.097)	0.026 (-0.088, 0.14)	-0.038 (-0.157, 0.081)	0.099 (-0.058, 0.256)
Postoperative sepsis	0.6	0.5	0.5	0.6	-0.066 (-0.208, 0.076)	-0.116 (-0.321, 0.09)	-0.105 (-0.299, 0.089)	-0.169[c] (-0.367, 0.028)	-0.078 (-0.302, 0.145)

Abbreviations: CI, confidence interval; DID, difference-in-differences; DVT, deep vein thrombosis; PE, pulmonary embolism; PSI, Patient Safety Indicator.

Notes: DID estimates are from linear probability models and can be interpreted as the pre-post percentage point difference between acquired and comparison hospitals in the percentage of stays that had the complication in the hospital. All models are adjusted according to the Quality Indicator software, with some exceptions, plus additional patient, hospital, and community characteristics (eMethods 3).

[a] The pre- and postmerger period descriptive data are based on all pre- and postmerger years available from each hospital, up to 10 years before the merger and 10 years after the merger. Sample sizes in the pre- and postmerger period are shown in eTable 7.

[b] The third, fourth, and fifth postmerger year models are separate models that include only hospitals with 3 or more, 4 or more, and 5 or more postmerger years of data, respectively.

[c] $P < 0.10$

Source: Agency for Healthcare Research and Quality, Healthcare Cost and Utilization Project, State Inpatient Databases, 2008–2018.

© 2021 Jiang HJ et al. *JAMA Network Open.*

eTable 8. Changes in Quality and Patient Safety for Stays at Hospitals That Merged and at Comparison Hospitals Assessed Using Logistic Regression Models

Quality indicator	Pre-post difference, by year, between merged and comparison hospitals DID estimate (95% CI) expressed as an odds ratio				
	Model 1		Model 2[b]	Model 3[b]	Model 4[b]
	1 year post	2 years post	3 years post	4 years post	5 years post
**Any IQI mortality**	0.86[e] (0.77 to 0.96)	0.85[d] (0.75 to 0.96)	0.83[e] (0.73 to 0.94)	0.80[e] (0.68 to 0.95)	0.77[e] (0.64 to 0.93)
AMI	0.75[e] (0.63 to 0.91)	0.76[e] (0.62 to 0.94)	0.76[d] (0.59 to 0.99)	0.66[e] (0.51 to 0.85)	0.76[c] (0.57 to 1.02)
Heart failure	0.83[d] (0.70 to 0.98)	0.89 (0.75 to 1.05)	0.77[e] (0.64 to 0.92)	0.80[d] (0.64 to 0.99)	0.74[d] (0.58 to 0.95)
Acute stroke	0.90 (0.74 to 1.10)	0.84 (0.66 to 1.06)	0.91 (0.73 to 1.14)	0.75[d] (0.58 to 0.97)	0.64[e] (0.46 to 0.90)
GI hemorrhage	0.77[c] (0.59 to 1.00)	0.73[d] (0.55 to 0.97)	0.81 (0.59 to 1.11)	0.93 (0.67 to 1.29)	0.88 (0.62 to 1.24)
Hip fracture	1.03 (0.77 to 1.38)	0.95 (0.71 to 1.28)	1.33[c] (0.98 to 1.82)	0.89 (0.59 to 1.35)	1.02 (0.64 to 1.62)
Pneumonia	0.93 (0.80 to 1.09)	0.9 (0.75 to 1.06)	0.82[c] (0.67 to 1.01)	0.85 (0.68 to 1.08)	0.77[c] (0.59 to 1.02)
**Any PSI complication**	0.96 (0.81 to 1.13)	0.94 (0.79 to 1.13)	0.99 (0.82 to 1.21)	0.85 (0.68 to 1.07)	0.94 (0.74 to 1.19)

Abbreviations: AMI, acute myocardial infarction; CI, confidence interval; DID, difference-in-differences; GI, gastrointestinal; IQI, Inpatient Quality Indicator; PSI, Patient Safety Indicator

Notes: All models are adjusted according to the Agency for Healthcare Research and Quality's Quality Indicator software, with some exceptions, plus additional patient, hospital, and community characteristics (eMethods 3). For both the intervention and comparison groups, the rate of the IQIs and PSIs generally decreased from the pre- to the postmerger period. Thus, a DID estimate below 1 indicates that the decrease in the intervention group was greater than the decrease in the comparison group.

[a] The pre- and postmerger period descriptive data are based on all premerger and postmerger years available from each hospital, up to 10 years before the merger and 10 years after the merger. Sample sizes in the pre- and postmerger periods are shown in the Supplement (eTable 10).

[b] The third, fourth, and fifth postmerger year models are separate models that include only hospitals with 3 or more, 4 or more, and 5 or more postmerger years of data, respectively.

[c] $P < 0.10$

[d] $P < 0.05$

[e] $P < 0.01$

Source: Agency for Healthcare Research and Quality, Healthcare Cost and Utilization Project, State Inpatient Databases, 2008–2018.

© 2021 Jiang HJ et al. *JAMA Network Open.*

eTable 9. Changes in Quality and Patient Safety for Stays in the Catchment Areas of Hospitals That Merged and in the Catchment Areas of Comparison Hospitals

Quality indicator	Pre-post difference, by year, between merged and comparison hospitals DID estimate (95% CI) in percentage points				
	Model 1		Model 2[b]	Model 3[b]	Model 4[b]
	1 year post	2 years post	3 years post	4 years post	5 years post
**Any IQI mortality**	0.028 (-0.086 to 0.142)	-0.024 (-0.147 to 0.100)	-0.035 (-0.160 to 0.089)	-0.040 (-0.160 to 0.080)	-0.070 (-0.219 to 0.079)
AMI	-0.246 (-0.728 to 0.236)	-0.147 (-0.614 to 0.321)	-0.262 (-0.742 to 0.218)	-0.466[c] (-1.012 to 0.080)	-0.583 (-1.198 to 0.033)
Heart failure	-0.411[d] (-0.748 to -0.074)	-0.094 (-0.432 to 0.244)	-0.410[d] (-0.773 to -0.047)	-0.176 (-0.592 to 0.239)	-0.225 (-0.743 to 0.293)
Acute stroke	0.090 (-0.526 to 0.706)	-0.215 (-0.889 to 0.459)	-0.171 (-0.782 to 0.439)	-0.783[d] (-1.563 to -0.004)	-0.165 (-0.983 to 0.654)
GI hemorrhage	-0.212 (-0.593 to 0.169)	-0.298 (-0.689 to 0.094)	-0.199 (-0.582 to 0.184)	-0.007 (-0.426 to 0.412)	0.023 (-0.490 to 0.537)
Hip fracture	-0.016 (-0.536 to 0.504)	-0.281 (-0.788 to 0.226)	-0.026 (-0.556 to 0.503)	-0.141 (-0.755 to 0.473)	-0.123 (-0.832 to 0.585)
Pneumonia	-0.097 (-0.474 to 0.280)	-0.131 (-0.505 to 0.242)	-0.106 (-0.533 to 0.322)	0.142 (-0.330 to 0.614)	-0.162 (-0.709 to 0.384)
**Any PSI complication**	-0.281 (-1.345 to 0.782)	-0.334 (-1.433 to 0.765)	0.696 (-0.339 to 1.731)	0.213 (-0.989 to 1.415)	0.192 (-1.045 to 1.429)

Abbreviations: AMI, acute myocardial infarction; CI, confidence interval; DID, difference-in-differences; GI, gastrointestinal; IQI, Inpatient Quality Indicator; PSI, Patient Safety Indicator

Notes: DID estimates are from linear probability models and can be interpreted as the pre-post percentage point difference between merged and comparison hospitals in the percentage of stays that died in the hospital (IQI) or with complication (PSI). All models are adjusted according to the Agency for Healthcare Research and Quality's Quality Indicator software, with some exceptions, plus additional patient, hospital, and community characteristics (eMethods 3).

[a] The pre- and postmerger period descriptive data are based on all premerger and postmerger years available from each hospital, up to 10 years before the merger and 10 years after the merger.

[b] The third, fourth, and fifth postmerger year models are separate models that include only hospitals with 3 or more, 4 or more, and 5 or more postmerger years of data, respectively.

[c] $P < 0.10$

[d] $P < 0.05$

Source: Agency for Healthcare Research and Quality, Healthcare Cost and Utilization Project, State Inpatient Databases, 2008–2018.

© 2021 Jiang HJ et al. *JAMA Network Open.*

eTable 10. Premerger and Postmerger Period Sample Sizes

Discharge sample	Discharges, premerger period		Discharges, postmerger period		Hospitals	
	Merged hospitals	Comparison hospitals	Merged hospitals	Comparison hospitals	Merged	Comparison
**Denominator, any IQI, N**	**303,747**	**461,092**	**269,881**	**444,902**	**172**	**266**
AMI	20,971	41,326	28,544	46,777	171	266
Heart failure	84,946	116,530	81,016	120,534	172	266
Acute stroke	30,344	44,259	28,174	44,390	171	266
GI hemorrhage	40,098	55,153	33,976	52,959	172	266
Hip fracture	21,757	34,984	22,077	36,898	165	260
Pneumonia	105,631	168,840	76,094	143,344	172	266
**Denominator, any PSI, N**	**175,970**	**278,070**	**151,484**	**259,891**	**162**	**242**
Perioperative hemorrhage	171,159	270,335	147,421	253,160	162	241
Postoperative respiratory	157,539	244,718	134,471	228,825	161	242
Perioperative PE/DVT	175,831	277,779	151,393	259,634	161	242
Postoperative sepsis	163,562	259,088	141,047	242,731	162	241

Abbreviations: AMI, acute myocardial infarction; DVT, deep vein thrombosis; GI, gastrointestinal; IQI, Inpatient Quality Indicator; PE, pulmonary embolism; PSI, Patient Safety Indicator.

Note: Up to 10 years of premerger period and 10 years of postmerger period data are included in the study, depending on the year of the merger.

Source: Agency for Healthcare Research and Quality, Healthcare Cost and Utilization Project, State Inpatient Databases, 2008–2018.

eFigure 1. Mean Annual Number of Stays for Elective Procedures Across Hospitals and Percentage of All Stays With Any PSI Complication

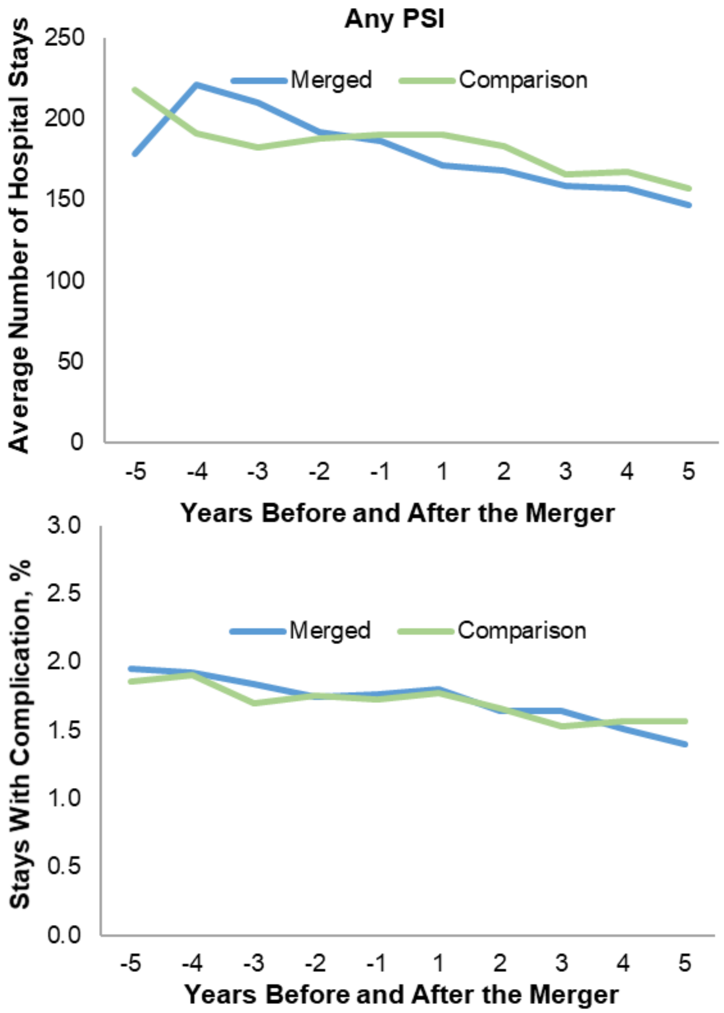

Abbreviation: PSI, Patient Safety Indicator.

Note: All hospitals were required to have 2 years of pre- and 2 years of postmerger data. The set of discharges at hospitals included in the 3-, 4-, and 5-year averages before and after the merger is for a different set of hospitals than the full sample.

Source: Agency for Healthcare Research and Quality, Healthcare Cost and Utilization Project, State Inpatient Databases, 2008–2018.

© 2021 Jiang HJ et al. *JAMA Network Open.*

eFigure 2. Hospital Scatter Plots of Inpatient Volume and In-Hospital Mortality Rates for the IQIs

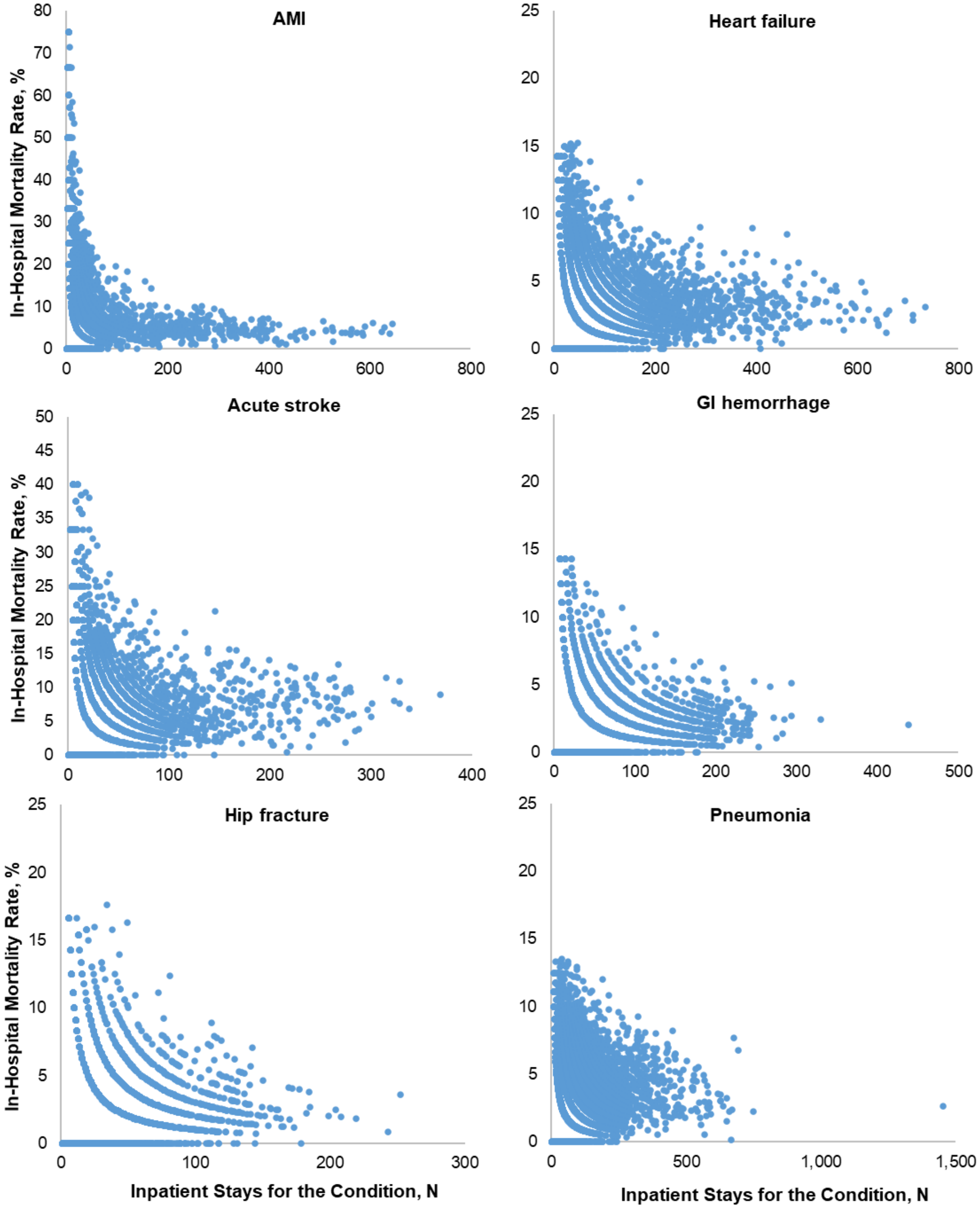

Abbreviation: AMI, acute myocardial infarction; GI, gastrointestinal; IQI, Inpatient Quality Indicator.

Note: Each panel includes one data point for each merged and comparison hospital in each year pre- and postmerger.

Source: Agency for Healthcare Research and Quality, Healthcare Cost and Utilization Project, State Inpatient Databases, 2008–2018.

© 2021 Jiang HJ et al. *JAMA Network Open.*
